# Real-time tuning of plasmonic nanogap cavity resonances through solvent environments

**DOI:** 10.1515/nanoph-2024-0749

**Published:** 2025-07-04

**Authors:** Eunso Shin, Rachel E. Bangle, Maiken H. Mikkelsen

**Affiliations:** Department of Electrical and Computer Engineering, 3065 Duke University, Durham, NC, 27708, USA; Department of Chemistry, North Carolina Agricultural & Technical State University, Greensboro, NC, 27405, USA; Department of Physics, Duke University, Durham, NC, 27708, USA

**Keywords:** metasurface, dynamic tuning, optofluidics, nanoparticle, visible to near-IR

## Abstract

Nanogap cavity metasurfaces – an array of metallic nanoparticles separated from a metal plane by a nanometer-scale dielectric material – can manipulate electromagnetic waves across a wide wavelength range. Through this, they can profoundly modify the optical processes of molecules and materials relevant to quantum communications, photocatalysis, and optoelectronics. Interactions between nanocavities and light, however, require overlap between the cavity resonance and the energy of the incident photon or optical transition, demanding labor-intensive fabrication of bespoke metasurfaces for each desired application. Here, we dynamically tune the resonance wavelength of nanogap cavity metasurfaces by modulating the refractive index of the surrounding medium using solvents. We achieve precise, reversible, and broadband resonance control for narrow nanogap cavity resonances (full width half max <500 nm) over a range of 1–5 µm, while maintaining high absorption efficiency (60–98 %). Resonance tuning up to 300 nm for a single metasurface was achieved by changing the dielectric environment from air to solvents with controlled refractive indexes *n* = 1.3–1.7 without any discernable metasurface degradation. This opens new possibilities for applications in optical sensing with significantly increased nanofabrication tolerances, such as tunable photonic devices and adaptive optical systems, where precise control over light–matter interactions is critical.

## 1 Introduction

Nanogap cavity metasurfaces have garnered significant attention for their ability to manipulate light through structures with deeply subwavelength dimensions. These metasurfaces, which consist of a shaped metallic nanoparticle separated from a flat metallic mirror by a thin dielectric layer, efficiently absorb or scatter resonant light through coupled surface plasmon resonances of the two metal structures. These processes create intense, subwavelength electromagnetic fields localized in the space between the metal film and nanoparticle, here termed the “gap.” These fields influence the radiative processes and optical properties of integrated materials and molecules, making nanogap cavity metasurfaces powerful tools for a wide range of photonic applications [1], [2], [3], [4], [5], [6]. Typically, the optical properties of nanogap cavity metasurfaces are statically determined at the time of nanofabrication through nanoparticle shape and size, dielectric layer thickness, and material identities [2], [3]. In contrast, real-time tuning introduces active adjustability of metasurface optical properties, which can significantly relax nanofabrication tolerances and enable new device functionalities such as electro-optical modulation [7], [8], [9], [10], [11], [12], [13], [14], adaptive imaging [12], [15], [16], [17], or tunable beam steering [18], [19], [20]. Reported active tuning methods have allowed for modulation of metasurface behavior through application of external stimuli [21], such as voltages [7], [8], [9], [10], [11], [12], [13], [14], [17], temperature shifts [22], [23], [24], [25], and mechanical forces [26], [27], [28]. Electrical tuning has offered kilohertz to gigahertz switching speeds and broad tuning ranges up to nearly 1 μm, however it generally requires large gate voltages and additional fabrication steps [7], [8], [9], [10], [11], [12], [13], [14]. Metasurfaces which incorporate phase change materials have exhibited substantial tuning ranges up to 500 nm upon heating. However, precise temperature control within metasurfaces is challenging, and the system inherently requires stabilization time to achieve reliable performance control [22], [23], [24], [25]. In addition, surrounding solvent media have been demonstrated to tune the resonance and diffraction efficiency of a silicon pillar metasurface by modifying the refractive environment without altering the metasurface or necessitating additional nanofabrication steps [16], [29]. As such, real-time solvent tuning shows great promise for adaptable metasurfaces used in imaging [15], [16], sensors [30], [31], [32] and metrology systems [33]. While most optofluidic platforms have achieved ∼150 nm resonance tuning of metasurfaces operating in the visible portion of the electromagnetic spectrum [15], [29], [32], [33], relatively little attention has been given to studies in the infrared wavelength range.

Here, we demonstrate real-time resonance tuning of a nanogap cavity metasurface via solvent immersion and exchange. This method achieves metasurface resonance tuning up to 300 nm while maintaining 80–90 % of the initial absorption efficiency. By using a variety of nanogap cavity metasurfaces, this method enables both precise and extensive tuning across broadband wavelengths with initial resonances ranging from 800 nm to 5 μm. In addition, this method demonstrates high reversibility, as resonance deviations less than 5 nm were observed upon repeated cycling of the solvent environment. This technique shows great promise for applications which require precise, extensive, and repeatable tuning, such as optical detectors, tunable photonic devices, and real-time adaptive optical modulators for next-generation photonic technologies. Further, the fact that the “gap” is spatially separated from the surrounding dielectric environments provides an innate protection of delicate optically active materials from potentially reactive solvents. This makes these solvent-tunable nano-gap cavities excellent candidates for real-time control of light–matter coupling using chemically-sensitive materials.

## 2 Results and discussion

### 2.1 Solvent environments to tune metasurface resonance

**Figure 1:**
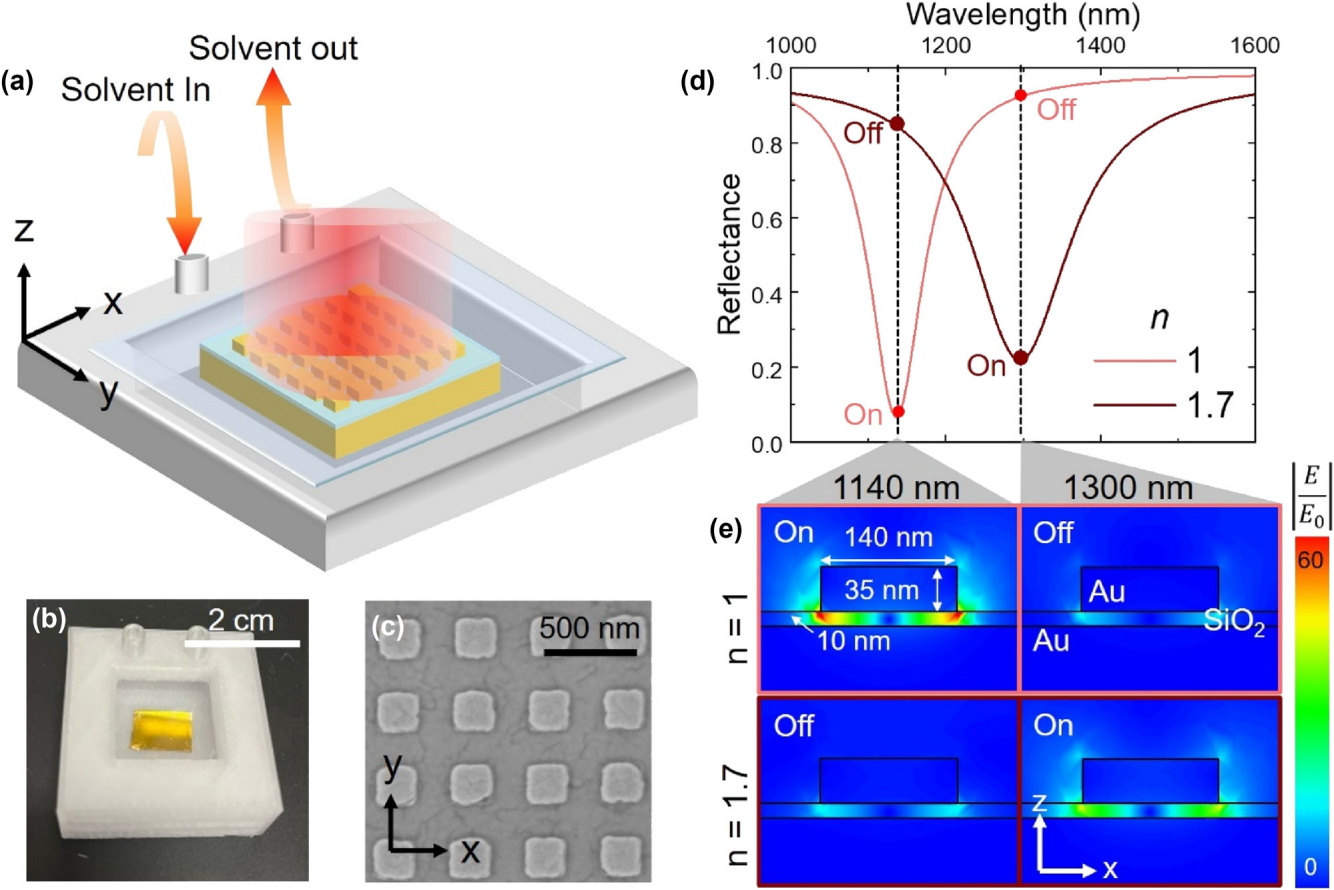
Geometric and dielectric control of resonant absorption in nano-gap metasurfaces. (a) Schematic representation of the metasurface sample in the flow cell. (b) Photographic image of the device flow cell. (c) SEM image of the metasurface elements. (d) COMSOL simulation of reflectance spectrum of the metasurface and (e) electric field at 1,140 and 1,300 nm for two refractive indexes (1 and 1.7) of surrounding medium. “On” and “off” refer to on- and off-resonance wavelengths.

Metasurfaces consisting of arrays of nanoscale patch antennas were constructed from square gold nanoparticles separated from a flat gold film by a thin dielectric spacer. The gold nanoparticles were square blocks with a height of 35 nm and side lengths (L) ranging from 120 to 1,100 nm, and nanoparticle arrays had a pitch of twice the side length. The dielectric spacer consisted of a SiO_2_ layer with thickness of 10, 20, or 40 nm deposited using plasma-enhanced chemical vapor deposition (PECVD) (Figure 1). As reported previously, the antenna array formed a metasurface which exhibited a highly efficient absorptive mode corresponding to the coupled plasmonic oscillations of the nanostructure and gold film. This results in significant light absorption at the resonance wavelength, where the resonance was determined by the side length of the gold nanoparticles and the SiO_2_ layer thickness. Metasurfaces with nanoparticle side lengths of 120–1,100 nm studied herein exhibited narrow resonances, with typical full width half max (FWHM) values ∼10–15 % of the resonance wavelength, across a broad spectral range from 1 µm to 5 μm (Fig. S1).

Each metasurface was fully immersed in a solvent environment established within a flow cell (1.5 cm × 1.5 cm × 1 cm) made from polypropylene using a 3D printer (Bambu lab x1-carbon). The solvent flow cell included two channels to facilitate solvent exchange, and a cover glass formed a window to ensure a uniform solvent thickness of 1 mm. The first channel was used to introduce solvents into the flow cell. After reflectance measurements, the second channel was used to remove the solvent. To wash away residue of the previous solvent, the subsequent solvent was flowed through the cell for 30 s before refilling the cell with fresh solvent. This cell enabled precise, real-time modulation of the refractive index surrounding the metasurface through exchange of commercial solvents (Cargille-Sacher) which act as refractive index standards (Figure 1a, Fig. S2).

Finite element simulations were used to predict the influence of the solvent refractive index (*n*, measured at 589 nm) on the metasurface absorption resonance (Figure 1d). The simulated absorbance of a metasurface with nanoparticle side lengths of 140 nm in air (*n* = 1) showed a maximum absorption (∼93 %) at 1,140 nm, while the same structure in a simulated solvent environment of *n* = 1.7 exhibited a maximum absorption at 1,300 nm (Figure 1d). As the refractive index near the nanostructure changed, it effectively changed the optical path length and the coupling conditions of the incident light. This caused the metasurface resonance frequency to decrease with higher *n* solvents according to:

$$\omega_{\text{res}}=\frac{\omega_{\text{p}}}{\sqrt{1+2\varepsilon_{m}}}$$

Here, ω_res_ is the effective resonance frequency, ω_p_ is the free electron plasma frequency of the metasurface, and *ɛ*_*m*_ is the permittivity of the surrounding medium. The resonance wavelength was inversely related to the resonance frequency, *λ*_res_ = 2*πc*/ω_res_ [1], [2], [3], [4]. Simulations further showed that absorption of resonant light generated extreme, heterogeneous electric fields within the dielectric layer (Figure 1e), while non-resonant light resulted in little to no cavity field.

**Figure 2:**
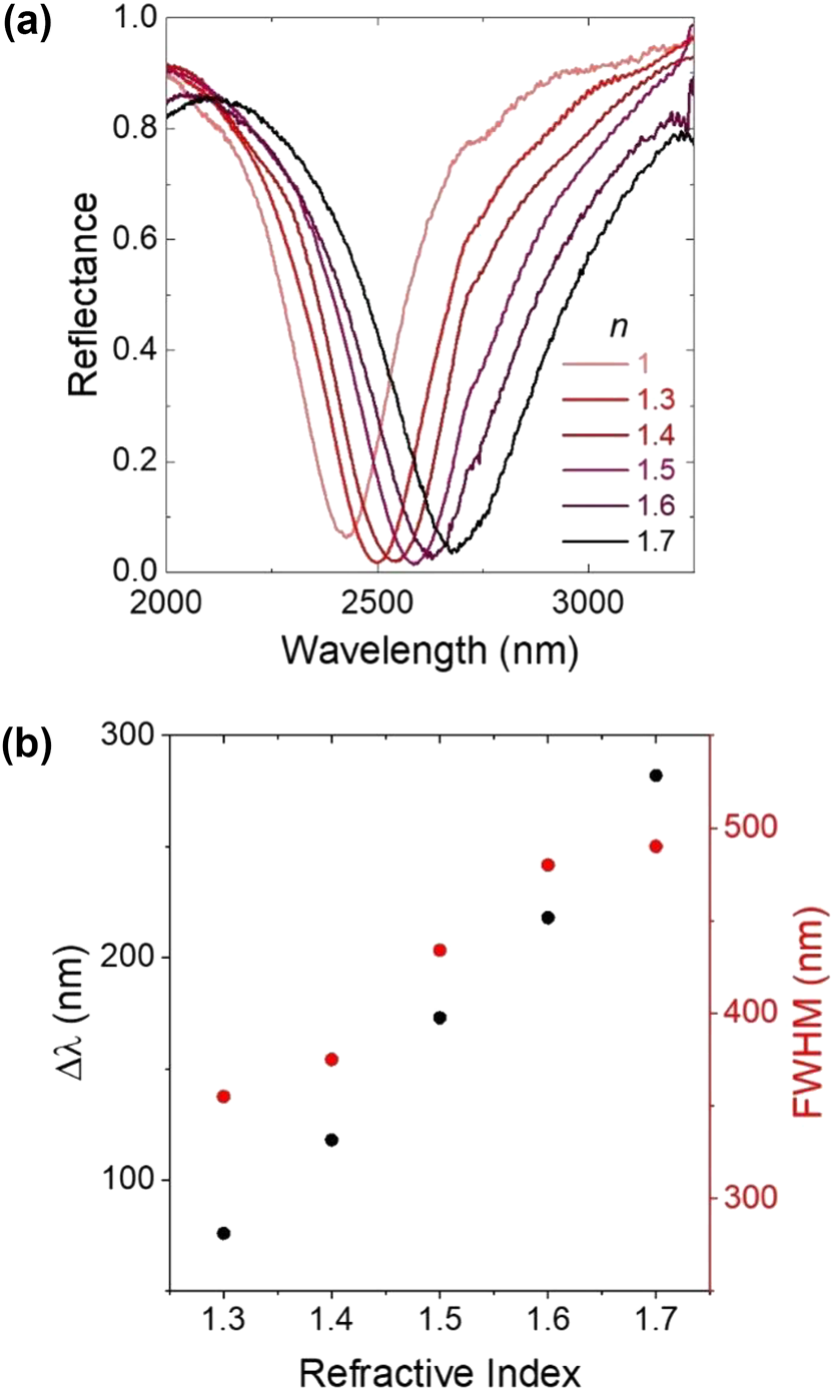
Resonance wavelength tuning via surrounding refractive index. (a) Experimental reflectance spectra of a metasurface (500 nm nanoparticle side length and 40 nm SiO_2_ thickness) showing the effect of increasing the refractive index of the surrounding medium, where higher indexes are represented by darker red colors. (b) Wavelength shift (black) and FWHM (red) of reflectance spectra with varying solvent refractive indexes.

Within the flow cell, the solvent environment was used to actively control the refractive index surrounding the metasurface, here consisting of square gold nanoparticles with 500 nm side lengths and a 40 nm thick SiO_2_ dielectric spacer. We utilized five different commercially available refractive index standards consisting of chlorofluorocarbon mixtures with refractive indexes between 1.3 and 1.7 measured at 589 nm (Fig. S2). The solvent was exchanged using the flow cell, and the reflectance spectrum was collected by a Fourier-transform infrared (FTIR) spectrometer (Bruker Invenio R). As shown in Figure 2a, the metasurface exhibited a resonance wavelength at 2,430 nm with 245 nm FWHM in air (*n* = 1). This FWHM was notably broader than predicted in simulations (150 nm), which likely results from small inhomogeneities in the electron beam lithography (EBL)-patterned metasurface. The side lengths of individual EBL-generated nanostructures exhibited a standard deviation of 4–7 nm, as quantified through scanning electron microscopy (SEM) images. As the refractive index increased, the resonance wavelength red-shifted linearly at a rate of 40–60 nm per 0.1 refractive index increment for a total shift of 280 nm from air to *n* = 1.7. This red-shifted tuning was accompanied by an increase in FWHM, though it should be noted that this increased FWHM becomes negligible when the spectra are plotted in wavenumber rather than wavelength (Fig. S3). Throughout the tuning, metasurface absorption remains consistently high (>95 %) (Figure 2).

### 2.2 Broadband wavelength tuning

**Figure 3:**
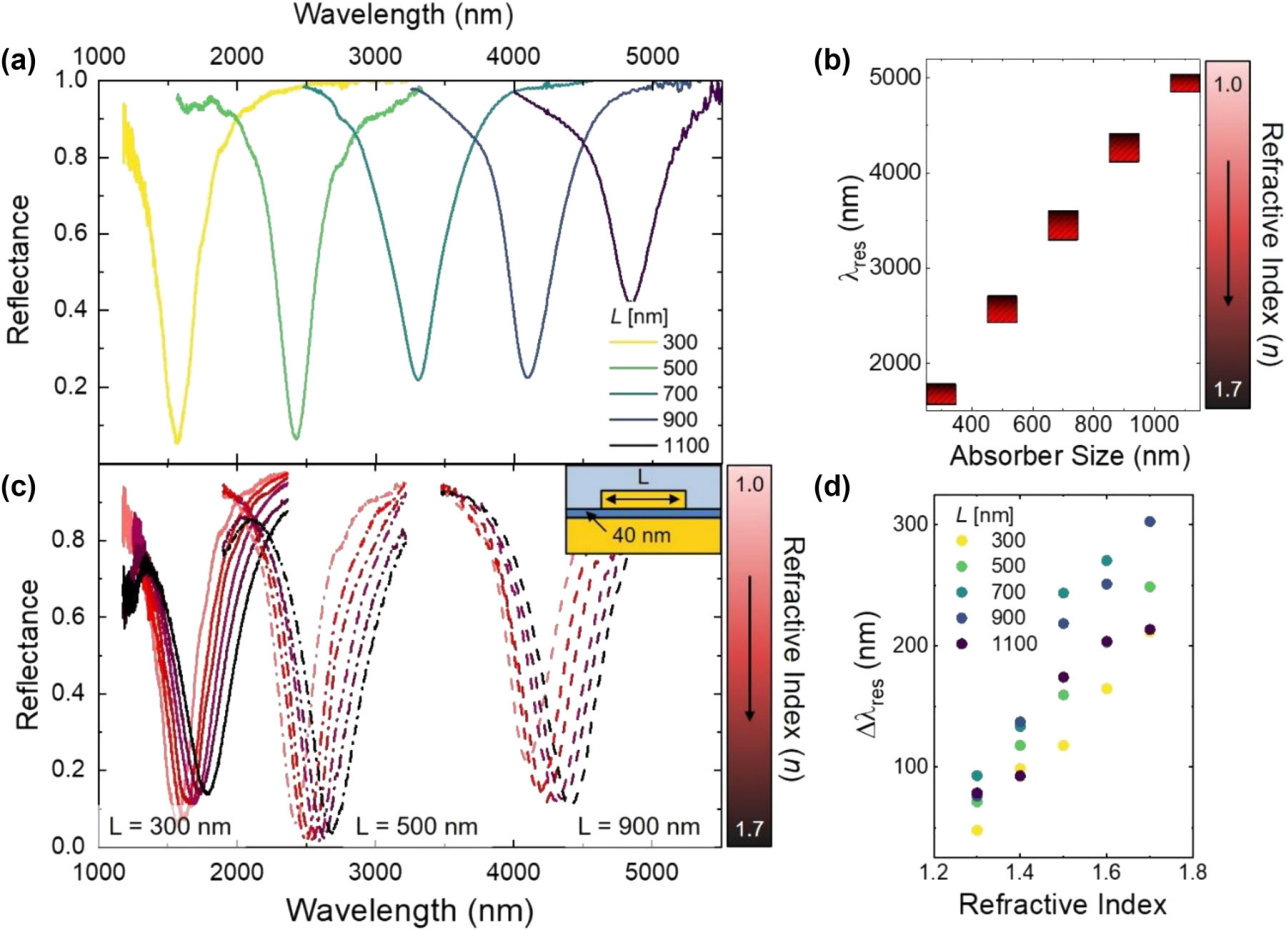
Real-time tuning across a broad spectral range. (a) Reflectance spectra of the metasurfaces of different side lengths (300–1,100 nm) with 40 nm SiO_2_ with longer lengths indicated by a darker color. (b) Red bars indicate resonance wavelength (λ_res_) tuning as a function of metasurface absorber size with refractive index change. Darker red colors represent higher refractive indexes. (c) Reflectance spectra of the metasurfaces of three side lengths (300, 500, 900 nm) with 40 nm SiO_2_ thickness indicated with solid, dash-dotted, and dashed lines, showing the resonance wavelength shifting with changing solvent refractive index. Darker red lines represent higher refractive indexes. Inset is a schematic of the metasurface structure where L is as indicated. (d) Change in resonance wavelength as the refractive index increases from 1 to the indicated refractive indexes. Darker dots indicate a larger nanoparticle side length (L).

Similar real-time resonance tuning through solvent exchange was observed over a wide range of nanoparticle side lengths and dielectric layer thicknesses, which resulted in real-time tuning over a broad spectral range. Spectra in Figure 3a correspond to metasurfaces of 300, 500, 700, 900 and 1,100 nm side lengths with a 40 nm SiO_2_ thickness. Increased nanostructure side lengths resulted in dramatic red shifts in peak absorption wavelength from 1 µm to 5 µm. Red shifts in the resonance were accompanied by a systematic decrease in absorption efficiency, which has previously been demonstrated to result in decreased electric field intensities within the larger mode volumes underneath nanoparticles with larger side lengths [34]. Nanostructures with 300 nm side lengths absorbed 98 % of incident light at the peak resonance wavelength (1,560 nm), while nanostructures with 1,100 nm side lengths absorbed 65 % incident light at the peak resonance wavelength (5,000 nm).

Exchange of the solvent environment resulted in similar resonance tuning across metasurfaces with all side lengths studied herein. Metasurfaces with resonances in air of 1.56, 2.42, and 4.11 μm each exhibited significant red-shifts in solvent environments with refractive indexes 1.0 to 1.7 as predicted in Eq. 1 (Figure 3c). Metasurfaces exhibited resonance tuning ranges up to 300 nm from air to *n* = 1.7, which corresponded to ∼ 17 meV resonance shift per 0.1 refractive index increment (Fig. S3). This demonstrated how tailoring the absorber size and adjusting the refractive index of the medium actively tuned the resonance wavelength across a broad wavelength range. Despite this broadband tunability, solvent tuning was limited in spectral regions where the solvent itself demonstrated optical activity. Specifically, the commercial solvent standards utilized in this study absorbed significant light at wavelengths 3,300–3,600 nm and > 5,000 nm (Fig. S4). In these spectral ranges, competitive solvent absorption masked the metasurface absorption and either prevented or obscured measurable resonance tuning (Fig. S1). This limit suggests that alternative solvent mixtures with higher IR transparency might allow for resonance tuning over a truly arbitrary spectral range well into the mid- and long wave-infrared portions of the electromagnetic spectrum.

**Figure 4:**
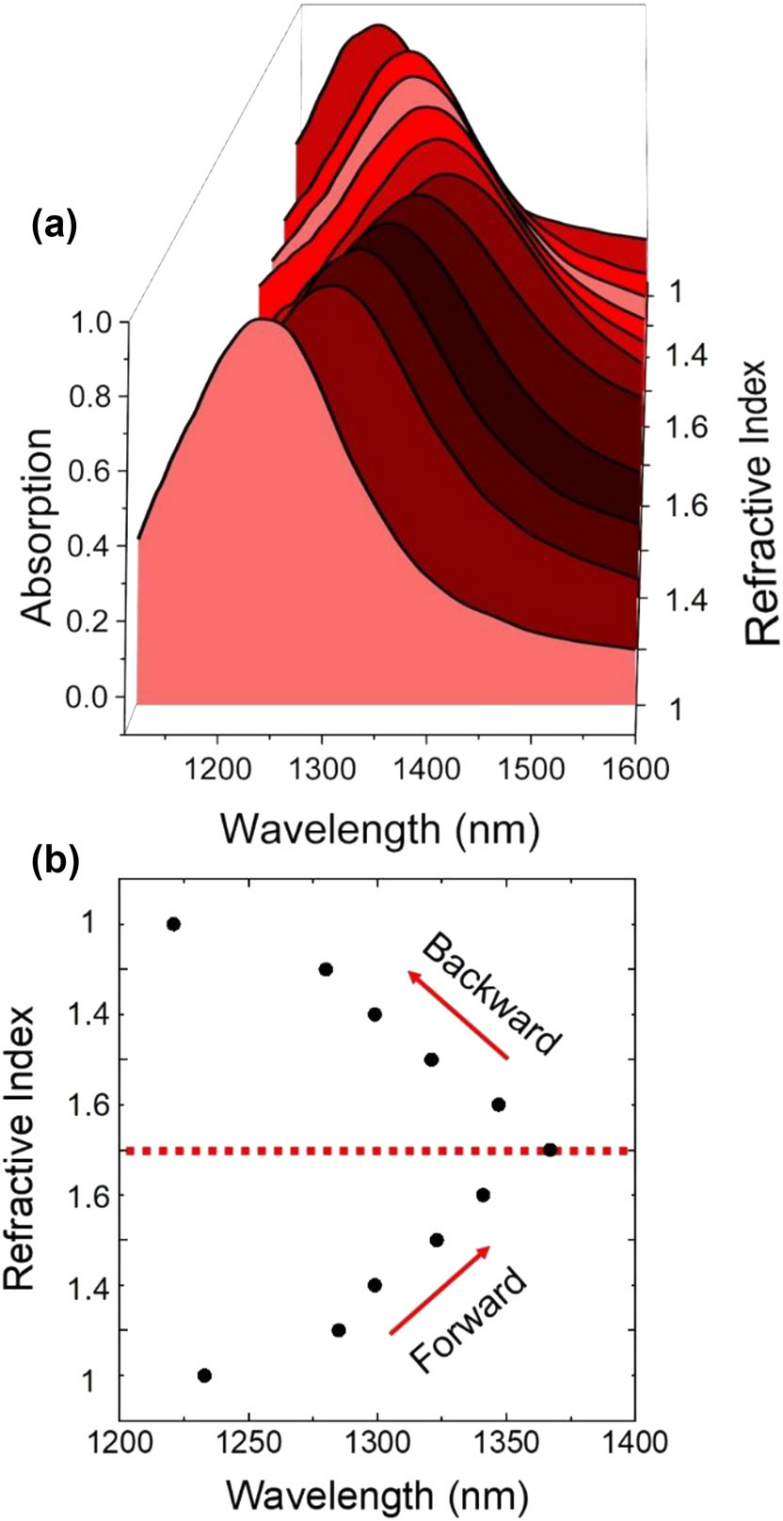
Reversibility of spectral shifts upon solvent exchange. (a) Hysteresis plot of absorption spectra showing the changes during a forward and backward sequence of solvent exchange. (b) Resonance wavelength shift as a function of refractive index, comparing the forward and backward sequence.

The metasurface resonance shifts due to changed solvent environment was reversible, exhibiting little or no hysteresis upon two solvent exchange cycles (Figure 4). Metasurface absorption spectra of a single sample were measured first upon solvent exchange from low to high refractive indexes and then from high to low refractive indexes. The resonance wavelength was observed to vary < 7 nm for a given refractive index between forward and reverse cycles, and spectra measured before and after the solvent exchange exhibited a minimal change in FWHM < 12 nm. A slight shift was detected at *n*=1 in the forward and backward sequences; we attribute this to small residual amounts of solvent trapped within the metasurface by capillary forces. However, by flushing the cell with additional solvent between measurements, the overall hysteresis remained below 7 nm. These results demonstrate the robustness and reliability of this tuning method, suggesting that the resonance wavelength can be consistently controlled with a high degree of precision.

### 2.3 Applications of nano-gap cavity microfluidics

The integration of microfluidic systems with nanophotonic devices and metasurfaces has been proposed as a promising tool for sensing, imaging, lasers, and more [32], [29]. A distinct advantage of integrating nano-gap cavity metasurfaces with microfluidics, however, is that cavity fields, which are localized within the gap layer, are spatially distinct from the solvent environment (Figure 1e). Though the environment surrounding the metal nanoparticle changes, the gap cavity itself, i.e. the dielectric material between the nanoparticle and metal film, does not come into direct contact with the solvent. This confers a distinct advantage to nano-gap cavities within microfluidic systems in contrast to more standard metasurfaces such as plasmonic lattice arrays, waveguides, gratings, and dielectric metasurfaces. Namely, the location of the field is protected from the potentially harsh and reactive solvents within the microfluidic device. As such, nano-gap cavity metasurfaces are uniquely well-suited to the integration of delicate materials such as quantum dots, transition metal dichalcogenides, small molecules, and polymer thin films into these fields. The broad microfluidic resonance tuning demonstrated herein could allow for the dynamic control of light–matter coupling, the Purcell effect, photothermal effects, or allowing otherwise forbidden of optical transitions while protecting the materials from potentially deleterious or complicating solvent interactions.

## 3 Conclusions

In this study, we investigated a dual tuning mechanism that combines the fabrication of metasurfaces with distinct resonance wavelengths and the modulation of the surrounding medium’s refractive index. By varying the metasurface design, we achieved resonance wavelengths ranging from 800 nm to 5 µm, each demonstrating a high absorption efficiency (60–98 %). Tuning the refractive index of the surrounding environment with solvents enabled broad resonance wavelength shifts up to 300 nm while maintaining high absorption efficiency. Typically, real-time tuning alone cannot achieve such a broad tuning range, but by integrating both passive and real-time tuning methods, we were able to achieve a wide tuning range for each resonance. Further, the structure of nano-gap cavity metasurfaces offers the advantage of spatially separating the solvents which cause the tuning from the cavity being tuned. This approach offers a promising method for dynamic tuning in applications such as optical sensing, modulators, light–matter coupling, and other tunable photonic devices [15], [16], [29], [30], [31], [32], [33].

## Supplementary Material

Supplementary Material Details
