# Supplementary material for: Real-time tuning of plasmonic nanogap cavity resonances through solvent environments
Source: Nanophotonics. 2025 Jul 4;14(23):3889–95. doi: 10.1515/nanoph-2024-0749 (PMC12617722; doi:10.1515/nanoph-2024-0749)
Supplement: Supplementary file 1 — Supplementary Material Details [file j_nanoph-2024-0749_suppl_001.pdf]

**Supplemental Information for:**

# Real-time tuning of plasmonic nanogap cavity resonances through solvent environments

*Eunso Shin*[1], *Rachel E. Bangle*[1,2] *and Maiken H. Mikkelsen*[1,3]*

[1] Department of Electrical and Computer Engineering, Duke University, Durham, NC 27708, USA

[2] Department of Chemistry, North Carolina Agricultural & Technical State University, Greensboro, NC 27405, USA

[3] Department of Physics, Duke University, Durham, NC 27708, USA

* Email: m.mikkelsen@duke.edu

**Sample Fabrication**

The metasurface samples were fabricated using metal evaporation, plasma enhanced chemical vapor deposition (PECVD), and electron beam lithography (EBL). A thin film of gold (75 nm) with ~1 nm RMS roughness was evaporated onto a Si substrate using an electron beam metal evaporator (CHA Industries Solution E-Beam). Subsequently, $SiO_2$ layers of varying thicknesses (10, 20, and 40 nm) were deposited using PECVD (Advanced Vacuum Vision 310). The thickness of the $SiO_2$ layers was verified with optical interferometry (Nanometry 210). To pattern the metasurface, EBL (Elionix ELS-7500 EX) was used. Polymethyl methacrylate (PMMA) 950A2 was applied to the substrate and spin-coated at 3000 rpm for 40 seconds to achieve a ~100 nm polymer film, followed by baking at 180°C for 2 minutes. Samples were exposed to an electron beam followed by development in a 1:3 ratio of Methyl isobutyl ketone (MIBK):isopropyl alcohol (IPA) solution for 45 seconds. 35 nm of gold and 5 nm of titanium were deposited using an E-beam metal evaporator, with titanium acting as an adhesion layer. EBL patterned absorbers' size was verified with scanning electron microscope (Apreo S by ThermoFisher Scientific) and showed around 4 -7 nm standard deviation.

**Reflectance Spectra**

The sample was prepared by applying solvent to the inlet channel at the flow cell covered with glass. Reflectance spectra were measured using a fourier transform infrared (FTIR) spectrometer (Bruker Invenio R). The white light beam size was controlled through an adjustable knife edge aperture, ensuring that the spot size remained consistent at 70 × 70 $\mu m^2$. The light propagated through the sample and was reflected back, passing through a beam splitter that directed the reflected light to the detector. Two detectors were used in this measurement: a silicon (Si) detector for the visible to near-infrared (NIR) range and a Mercury-Cadmium-Telluride (MCT) detector for the near-infrared to mid-infrared (MIR) range. After each measurement, the solvent was removed by the outlet channel of the flow cell. For washing out the residue solvent, the next solvent was flowed through the inlet channel. Reflectance spectra were fitted using a Lorentzian function based on a dipole approximation of a surface plasmon resonance which assumes the plasmonic response behaves as a damped harmonic oscillator.

**Simulations**

To investigate the principle of refractive index tuning in the surrounding medium, we employed Finite Element Simulations via COMSOL Multiphysics, utilizing the Wave Optics Module with the Electromagnetic Waves, Frequency Domain. The model consisted of a single gold nanostructure, designed with 140 × 140 × 35 nm with periodic boundary conditions in the horizontal direction to simulate a metasurface. In the vertical direction, we applied a Perfectly Matched Layer (PML) to absorb outgoing waves and prohibited boundary reflections. The refractive index of the surrounding medium was varied according to the manufacturer's dataset (Fig. S2), and used to assess the influence on the optical response of the metasurface.

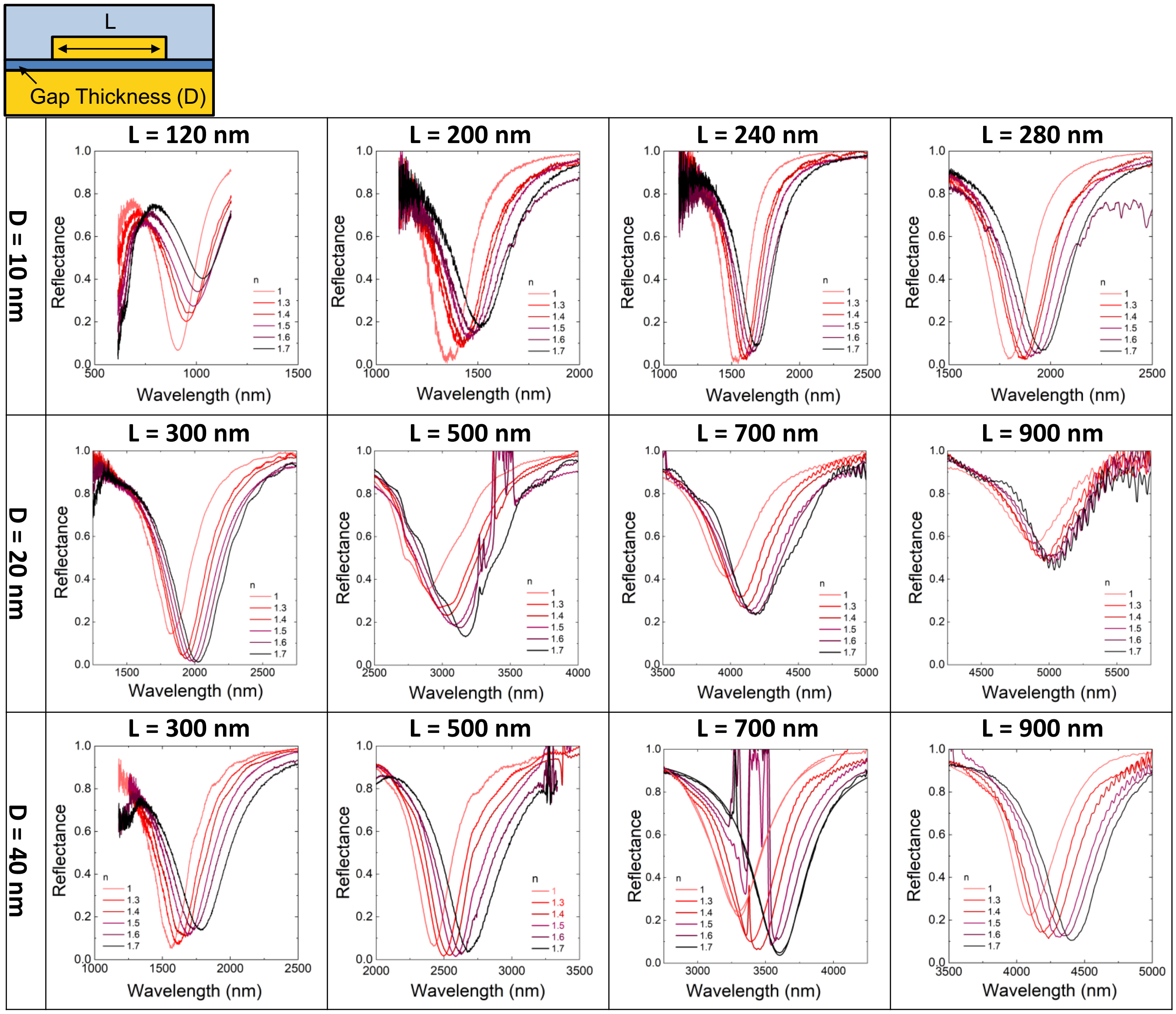


***Figure S1:*** *Reflectance spectra of different metasurface structures with changing solvent refractive indices. A few metasurface (500 nm side length with 20 nm gap thickness, 700 nm side length with 40 nm gap thickness) resonance wavelengths show a large peak around 3.5 μm. This is because of the high absorption from solvents. Inset is a schematic of metasurface, indicating a gap thickness (D) and side length (L).*

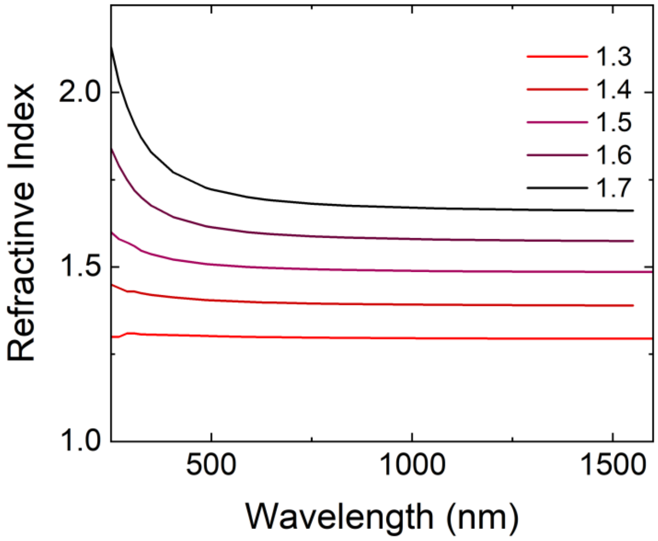


***Figure S2:*** *Real refractive index values of solvent standards as a function of wavelength, with darker red colors representing higher refractive indices solvents. These data are provided from Cargille-Sacher Laboratories Inc.*

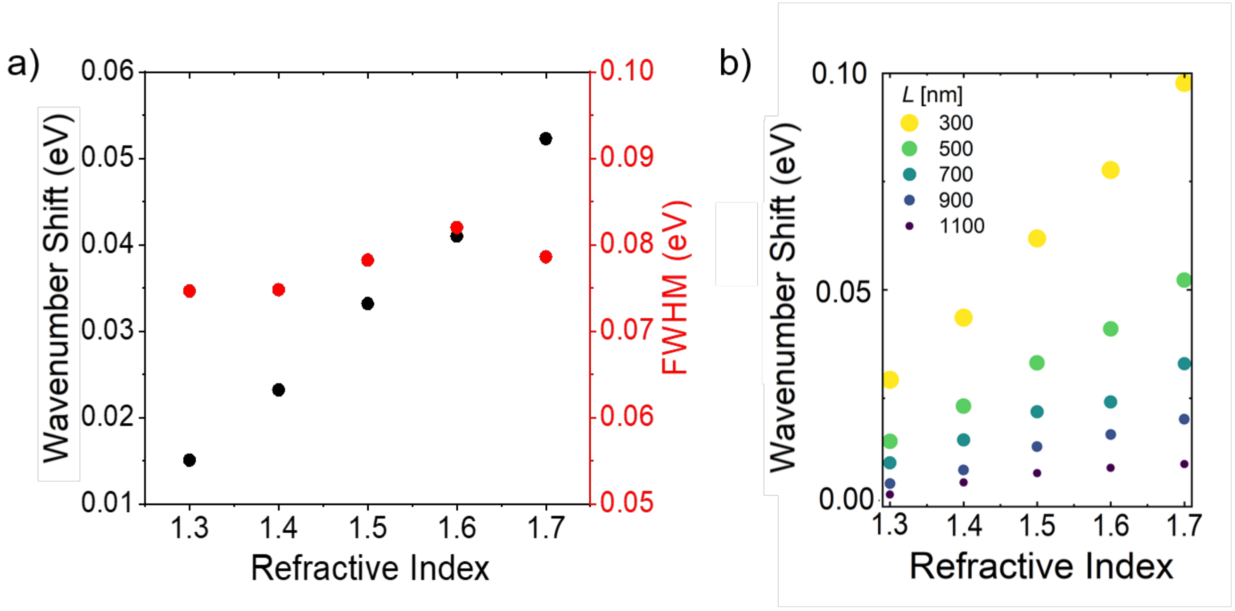


***Figure S3:*** *(a) Full width at half max (FWHM) (red) and resonance wavenumber shift (black) as the solvent refractive index increases from 1, with different refractive indices. (b) The resonance wavenumber shifts as the refractive index increases from 1, for various refractive index values with each metasurface. Darker dots indicate a metasurface consisting of nanoparticles with larger side lengths (L).*

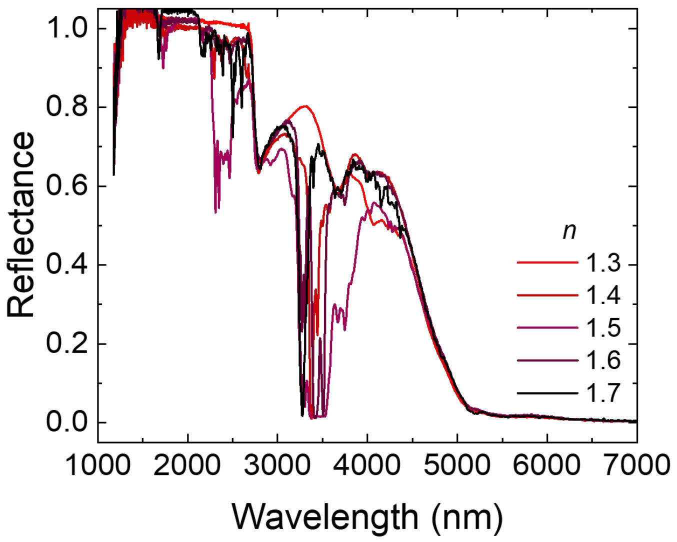


***Figure S4:*** *Reflectance spectrum of each solvent measured by FTIR***,** *where higher indices are represented by darker red colors***.** *Most of the solvents, except refractive index 1.3, have a high absorption value around 3.3 – 3.6 µm.*

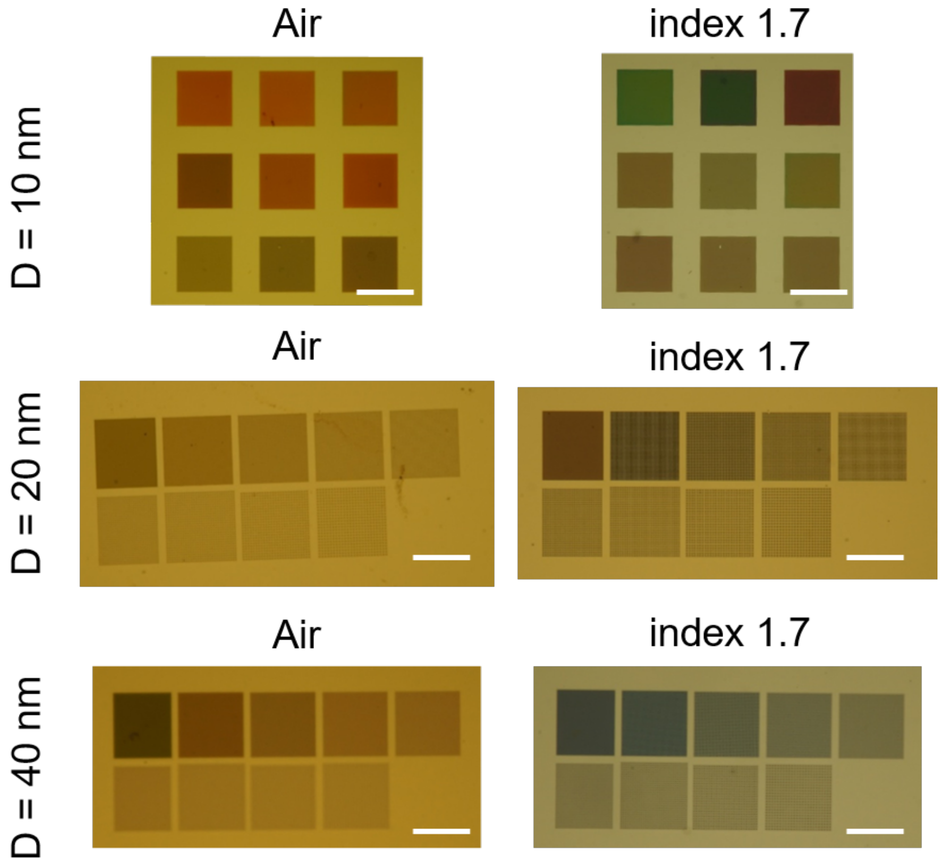


***Figure S5:*** *Optical microscopy images of different metasurfaces (gap thickness (D) = 10, 20, 40 nm) with two refractive indices (1.0 and 1.7) of surrounding medium. Scale bar is indicated 75 µm.*

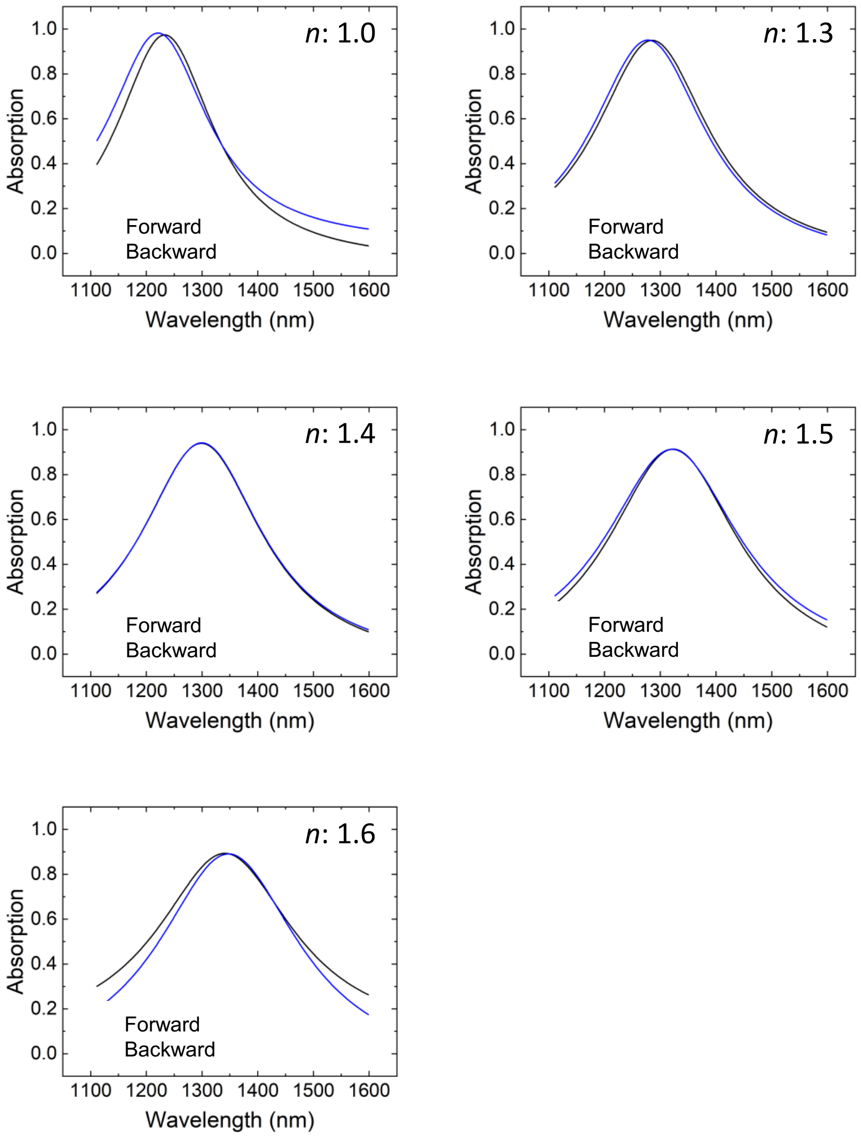


***Figure S6:*** *Absorption spectrum fitted with Lorentzian function of different refractive indices (n) with two different sequences, forward (black line) and backward (blue line).*

| | **Air** | **Index 1.3** | **Index 1.4** | **Index 1.5** | **Index 1.6** |
|---|---|---|---|---|---|
| Forward λ [nm] | 1233.1 | 1285.6 | 1299.2 | 1323.2 | 1341.3 |
| Backward λ [nm] | 1221.3 | 1278.2 | 1299.8 | 1321.6 | 1347.6 |
| Forward FWHM [nm] | 213.4 | 249.4 | 267.5 | 287.3 | 297.7 |
| Backward FWHM [nm] | 216.6 | 252.6 | 264.8 | 295.7 | 309.0 |

***Table S1:*** *Resonance wavelength and FWHM of each absorption spectrum extracted from Fig. S6.*
